# Bovine neutrophils form extracellular traps in response to the gastrointestinal parasite *Ostertagia ostertagi*

**DOI:** 10.1038/s41598-018-36070-3

**Published:** 2018-12-04

**Authors:** Jonatan Mendez, Donglei Sun, Wenbin Tuo, Zhengguo Xiao

**Affiliations:** 1 Department of Avian and Animal Sciences, University of Maryland, College Park, MD 20742 USA; 2 Department of Veterinary Medicine, University of Maryland, College Park, MD 20742 USA; 3 Animal Parasitic Diseases Laboratory, USDA/ARS, Beltsville, MD 20705 USA

## Abstract

*Ostertagia ostertagi* (OO) is a widespread parasite that causes chronic infection in cattle and leads to annual losses of billions of dollars in the cattle industry. It remains unclear why cattle are unable to mount an effective immune response despite a large influx of immune cells to the infected abomasal mucosa and draining lymph nodes. Neutrophils, the immune system’s first responders, have the capacity to release neutrophil extracellular traps (NETs) to contain various pathogens, including some parasites. In the present study, the mechanisms by which *O*. *ostertagi* influences bovine NET formation were investigated. *O*. *ostertagi* larval soluble extract (OO extract) was able to induce typical NETs by purified neutrophils *in vitro*, confirmed by co-localization of extracellular DNA with typical NET-associated proteins histone and neutrophil elastase (NE). Consistent with existing literature, inhibition assays demonstrated that these OO extract-induced NETs were dependent upon the enzymes NADPH oxidase and myeloperoxidase (MPO). Live OO stage 4 larvae (L4) stimulated neutrophils to form NETs similar to those induced by OO extract. Bovine neutrophils also released NETs in response to *Caenorhabditis elegans*, a free-living soil nematode, suggesting that bovine NET production may be a conserved mechanism against a broad range of nematodes. This is the first report demonstrating *O*. *ostertagi*-induced NET formation by bovine neutrophils, a potentially underappreciated mechanism in the early immune response against nematode infections.

## Introduction

*Ostertagia ostertagi*, a gastrointestinal (GI) nematode parasite of cattle and the causative agent of ostertagiasis, is considered one of the most economically most significant diseases in the cattle industry in temperate regions^1^. Ingested *O*. *ostertagi* third-stage larvae (L3) invade and take up residence in the gastric glands of the abomasum, causing cellular hyperplasia and leading to significant pathology^2,3^. Infection is attributed to an impaired protective immunity during infection^4,5^. The pasture is an intrinsic component of the life cycles of many livestock GI nematodes, including *O*. *ostertagi*, and serves as a natural reservoir for the infective L3 larvae; as such, grass-fed animals are at high risk of exposure to this parasite. In particular, infections can run unchecked in organic farming systems where anthelminthic drug usage is disallowed^6^. Moreover, while anthelmintics have been efficacious in the past, evidence of rapidly emerging anthelminthic drug resistance is mounting^7,8^. Thus, there is an urgent need for developing alternative nematode control strategies such as vaccination which could be used in combination with the anthelmintic treatment. However, the bovine immunity against ostertagiosis is poorly characterized. The overall goal of the present study was to understand the basic mechanisms of host-parasite interactions, in particular, neutrophil DNA release and neutrophil extracellular trap (NET) formation in response to *Ostertagia* infection in cattle.

*O*. *ostertagi* infection is known to elicit a mixed T helper (Th) cytokine response, distinct from the typical Th2 dominant response seen in model helminth infections^9^. In addition, the parasite has demonstrated a capacity to suppress immune responses such as lymphocyte proliferation and function^10,11^. Overall immune responses to *O*. *ostertagi* are poorly understood, and the early innate immune response to this parasite remains uncharacterized. As one of the first responders to infection and a source of local inflammation, neutrophils are recruited in significant numbers and may serve as the first line of defense against helminths^12^. While traditionally viewed as relatively simple and short-lived effector cells, recent findings on novel neutrophil functions have resulted in a shifting paradigm wherein neutrophils are implicated as an essential player in modulation of multiple early immune responses^13^. Importantly, the interactions between neutrophils and helminths such as *O*. *ostertagi*, which may represent a critical aspect of host-parasite interplay, have not been thoroughly investigated.

Neutrophils have several known effector functions against invading pathogens, including phagocytosis, release of antimicrobial molecules, production of reactive oxygen species (ROS), and release of neutrophil extracellular traps (NETs)^12^. NETs are large extracellular structures comprising a mesh of chromatin fibers containing an array of granule proteins such as neutrophil elastase (NE) and myeloperoxidase (MPO)^14,15^, which are capable of trapping and in some instances killing microbes. NETs have been characterized in several mammals including humans^14^, mice^16^, goats^17^, sheep^18^, and cattle^19–28^. While many studies have investigated the role of NETs on fungal^29^, bacterial^30^, viral^31^, and protozoan^32^ infections, relatively few have delved into the role of NETs in helminth infections. Neutrophils have been shown to release NETs in response to parasites^33^ such as *Neospora caninum*^23^, *Eimeria bovis*^26^ and *Haemonchus contortus*^34^, with this NET release shown to be MPO-, NE-, and ROS-dependent^23,26,34^. Recently, ROS-independent pathway has been reported^35,36^. These NETs have demonstrated a potential to entrap infectious nematodes such as *H*. *contortus*, suggesting NETs may be an important defense mechanism in mitigating other nematode parasite infections in general^34^. In addition to their direct interactions with invading pathogens, NETs can exert a direct enhancement^37^ or dampening^38^ of accompanying inflammatory responses depending upon the immunological context. The capacity for *O*. *ostertagi* to induce NET release, however, is unknown, and the role of these NETs, if released, may play in subsequent inflammation remains to be investigated.

The aim of the present study was to investigate *O*. *ostertagi*’s ability to induce NETs of bovine neutrophils and delineate the mechanisms that mediate this response, furthering our understanding of *O*. *ostertagi* pathogenesis and host responses and potentially revealing novel immunological treatment targets. The results of the present study suggest that *O*. *ostertagi* is capable of inducing NET release, without production of ROS. We also provide some evidence that this NET response may not be limited to specific nematode parasites such as *O*. *ostertagi* and may instead be a conserved response to any nematode encounter.

## Results

### OO extract induces NET release

**Figure 1:**
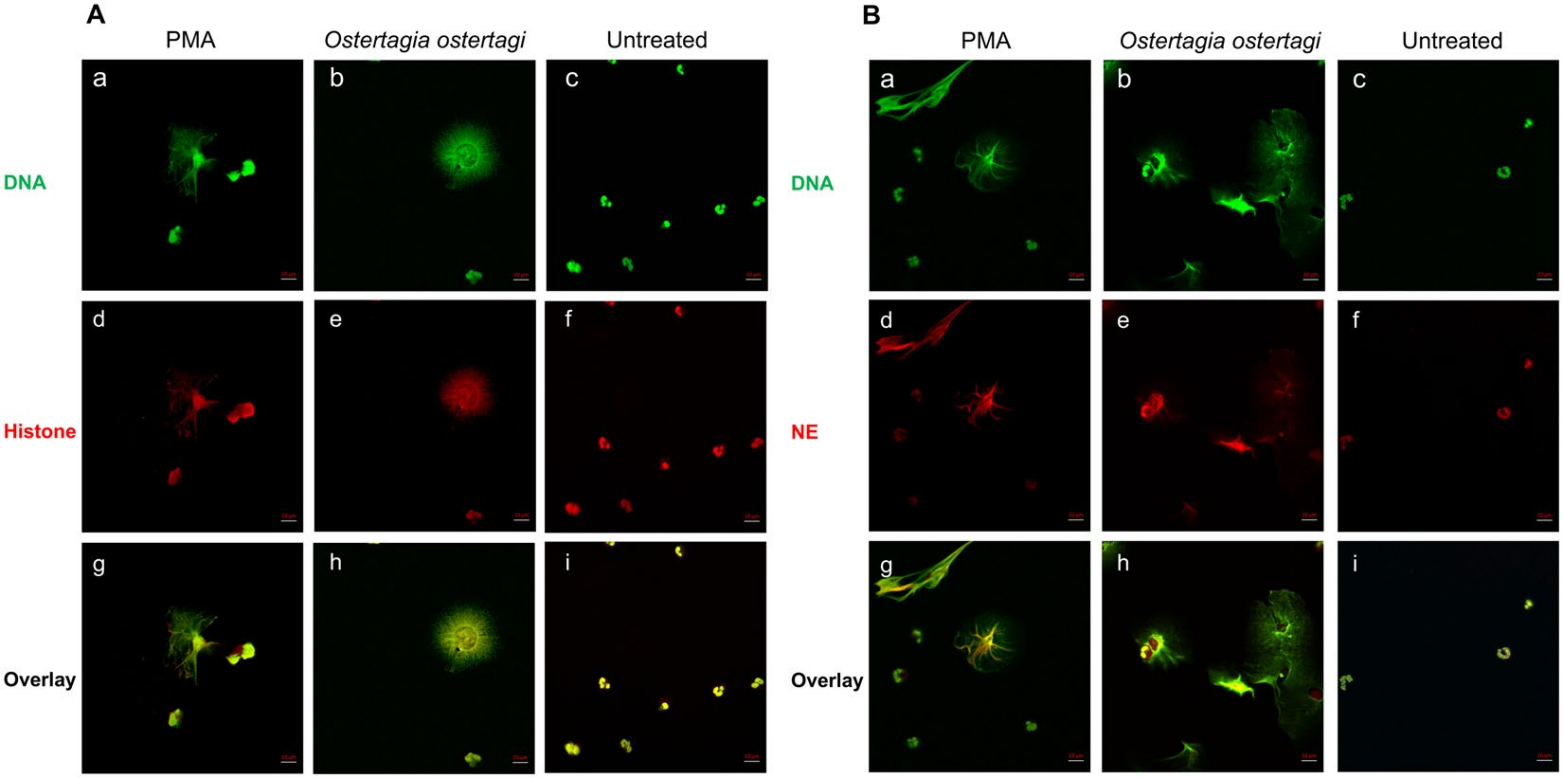
Co-localization of DNA with histone (H3) (**A**) and neutrophil elastase (NE) (**B**), in PMA stimulated (a,d,g), *Ostertagia ostertagi* (OO) extract stimulated (b,e,h) and unstimulated (c,f,i) bovine neutrophils and associated neutrophil extracellular trap structures. (a–c) DNA stained with Sytox Green (green). (d–f) Histone (H3) staining in (**A**), and NE staining in (**B**) within NET structures (red). (g–i) Overlay of NET-DNA with histone (**A**) or NE (**B**). The neutrophils were incubated with each stimulus for 3 hours before staining.

Studies on other ruminant parasites have demonstrated their ability to induce NETs in bovine neutrophils, thus we hypothesized that *O*. *ostertagi* could induce NET formation as well. OO extract was able to induce NETs with typical structures (Fig. 1). Sytox Green staining revealed that stimulation of bovine neutrophils with OO extract led to the release of a dense network of DNA fibers spreading outwards from the cell (Fig. 1A-b,B-b). These DNA structures co-localized with histone (Fig. 1A-h) and NE (Fig. 1B-h), two proteins widely used as markers of NETs, confirming the existence thereof. Unstimulated neutrophils showed normal multilobed nuclei and lacked extracellular DNA structures (Fig. 1A-c,B-c). Stimulation with the well-established NET inducer PMA led to the formation of similar large extracellular DNA structures (Fig. 1A-a,B-a) which also co-localized with histone (Fig. 1A-g) and NE (Fig. 1B-g), consistent with recent reports.

**Figure 2:**
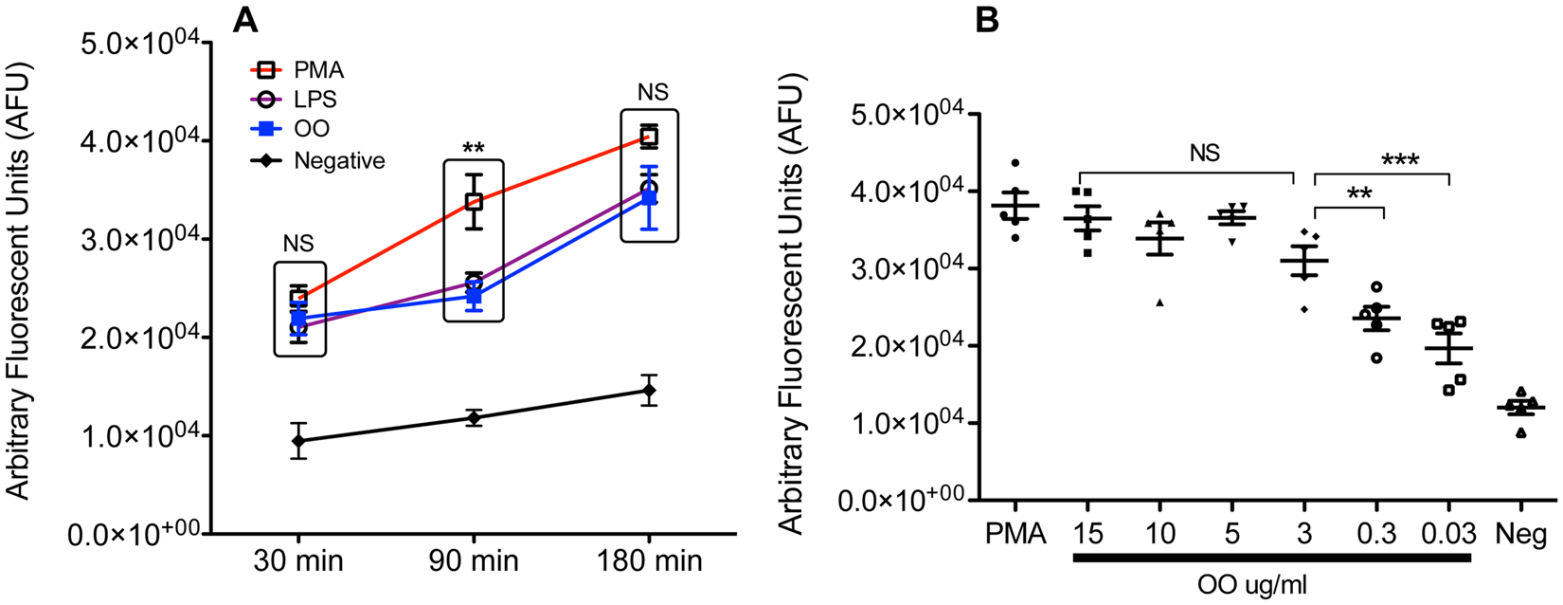
DNA release following Ostertagia ostertagi (OO) extract treatment of bovine neutrophils (**A**). Kinetics of PMA, LPS and OO extract-induced DNA release over a period of 3 hours. (**B**) Dose-dependent effect of OO extract on DNA release by neutrophils incubated for 3 hours. Experiments were performed using cells from five cattle. Data were expressed as mean +/− SEM. All data were analyzed by one-way ANOVA with Newman-Keuls Multiple Comparison Test. Data were representative of at least three experiments with similar results.

To further characterize the role of OO extract in NET induction, time- and dose-dependent responses were examined *in vitro*. The enhancement of extracellular DNA release at similar levels was detectable 30 min following incubation with PMA/LPS and OO extract (Fig. 2A), which was significantly higher in cells treated with PMA/LPS and OO extract than unstimulated cells (*p* < 0.01). The NET release was significantly greater up to 90 min post treatment in cells treated with PMA when compared to those induced by LPS or OO extract. In general, PMA appeared to induce higher average NETs than those stimulated by other treatments (Fig. 2A), which may reflect the ability of PMA to directly activate protein kinase C (PKC), whereas LPS and possibly OO extract may have to work through multi-stepped signal transductions^39^. NET release in response to OO extract illustrated a clear time-dependent pattern similar to that of LPS (Fig. 2A). With maximal NET release at 180 min for all treatments, all subsequent experiments were conducted for this amount of time. To further define the dose response of neutrophils to OO extract, purified neutrophils were incubated with different concentrations of OO extract for 3 h and displayed a dose-dependent NET release, with maximal production at 3 µg/mL of OO extract (Fig. 2B). All subsequent experiments were conducted using OO extract at this concentration (3 µg/mL). Treatment with DNase I lowered the detection to the level of controls (Fig. 2B), suggesting that the increase in detected DNA was mostly extracellular. The results were consistent with the detection of extracellular DNA in NETs induced by PMA, LPS, and OO in Fig. 1.

### OO extract-induced NET release is MPO-, NE-, and NADPH oxidase-dependent

**Figure 3:**
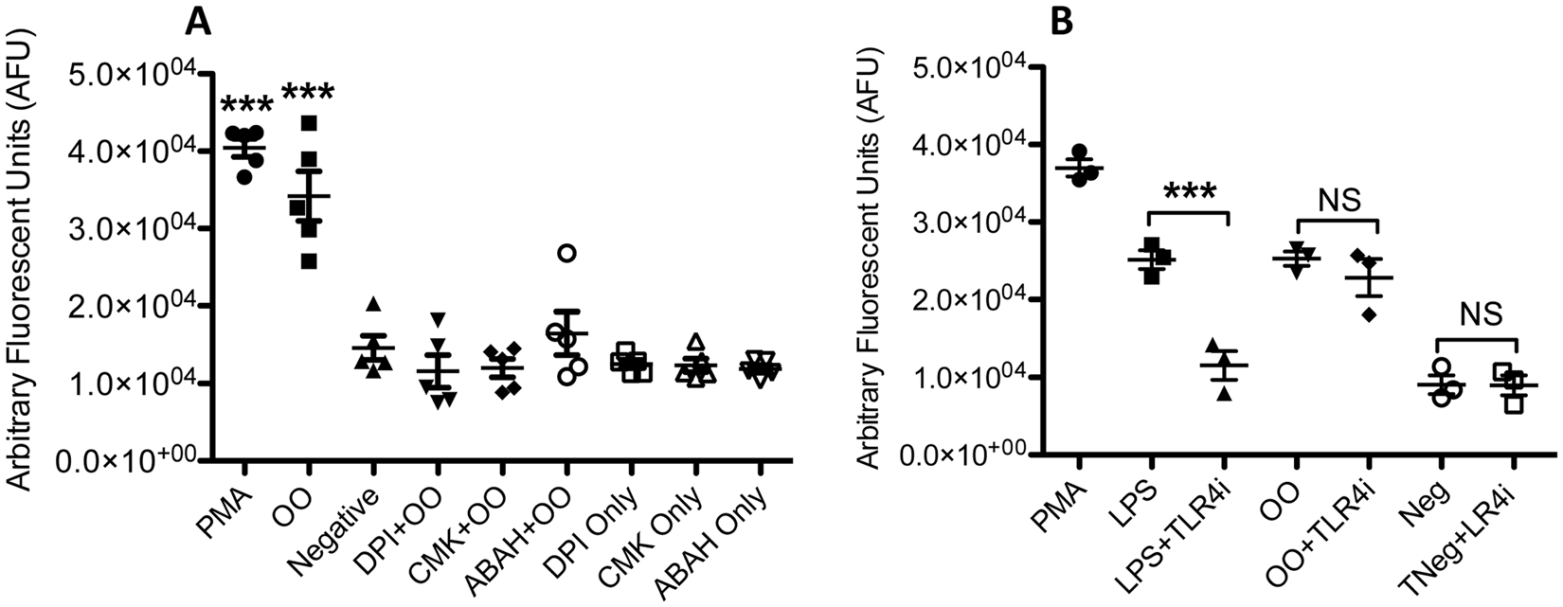
Blockage of Ostertagia ostertagi extract (OO)-triggered NETosis using inhibitors. (**A**) Inhibition of NADPH oxidase with DPI, NE with CMK, and MPO with ABAH, respectively. Data were expressed as mean +/− SEM from five cattle. (**B**) Inhibition of TLR4 with its inhibitor CLI-095 (TLR4i). Data were expressed as mean +/− SEM from triplicate using cells from one cattle. All data were analyzed by one-way ANOVA with Newman-Keuls Multiple Comparison Test. Data were representative of 3-4 experiments with similar results.

To further corroborate the characteristics of OO extract-induced NET release, inhibition experiments of these classical NET-associated molecules were performed using specific inhibitors for NE (CMK), MPO (ABAH), and NADPH oxidase (DPI). Neutrophils pre-incubated with each inhibitor demonstrated significant reductions in NET release following OO extract stimulation (*p* ≤ 0.01, Fig. 3A), confirming a definitive role for each of these enzymes in OO extract-induced NET release. As reported in other bovine parasite models of NET release, the ROS pathway and NADPH oxidase, in particular, appear to be an integral part of NET formation, although the role that specific ROS products have in mediating this response remains unclear. The OO extract contained whole parasite content, so may include some bacteria from their gut microbiota, which consist of many immune stimulating components, such as LPS from gram-negative bacterial walls, which binds to TLR4. To test this possibility, neutrophils were pretreated with TLR4 inhibitor before stimulated with OO extract. Inhibition of TLR4, with CLI-095, however, did not affect the NET release induced by OO extract (Fig. 3B), indicating OO extract-stimulated NET release is not dependent on TLR4. Polymixin B (PMB) has been reported to be effective in blocking LPS stimulation in human cells^40–42^. However, PMB seemed to enhance OO-induced NET release (although not significant), possibly due to direct induction of NET release by PMB itself (Supplementary Fig. 1); this has also been observed on human neutrophils^43^. PMB can accumulate in mitochondria leading to apoptosis^44,45^. Protein kinase C (PKC) is involved in PMA-induced NET formation^46,47^; however, PKC inhibition caused no significant changes in OO-stimulated NET formation (Supplementary Fig. 1), suggesting that NET release induced by OO may not induce signaling through PKC^46,47^.

### DNA in serum does not correlate with infection status

**Figure 4:**
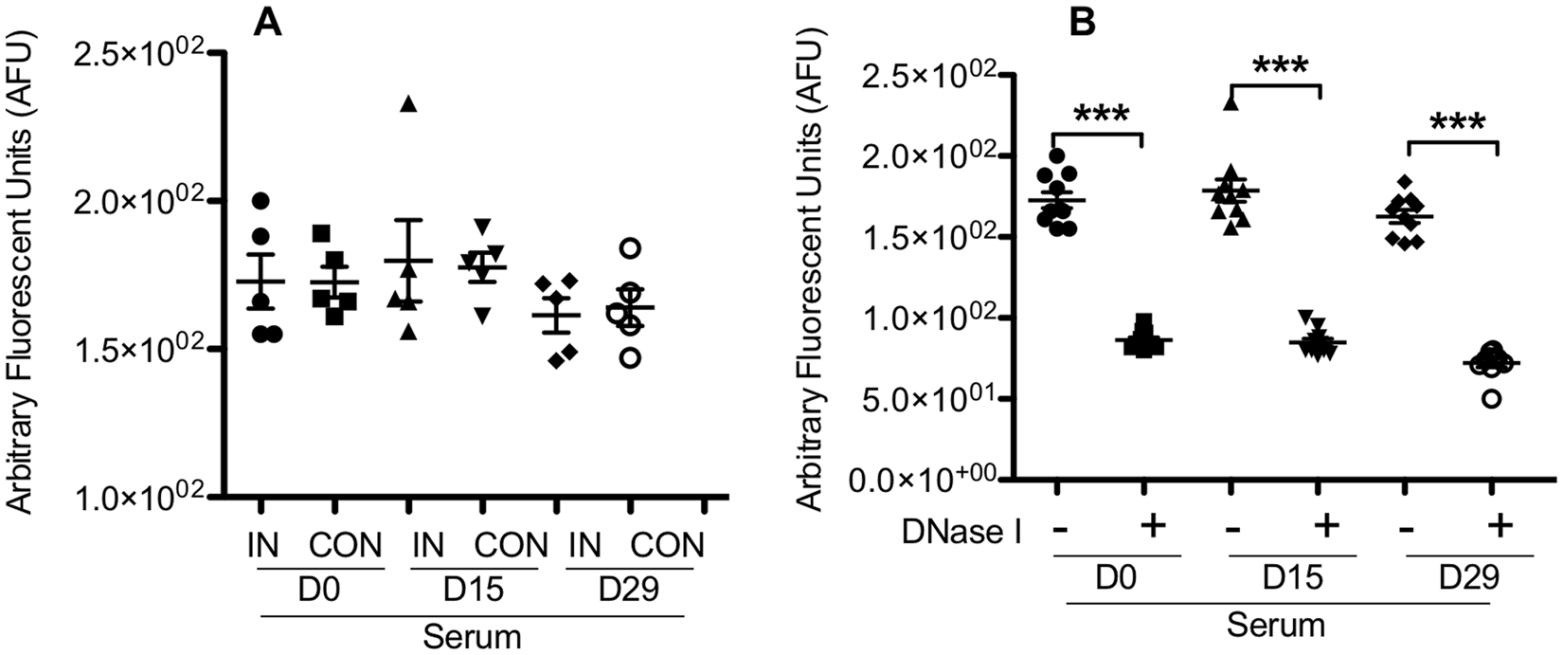
DNA concentrations in sera of cattle experimentally infected with Ostertagia ostertagi and uninfected controls on Days 0, 15, or 29 post infection. All data were analyzed by one-way ANOVA with Newman-Keuls Multiple Comparison Test. IN, infected; CON, control.

NETs are formed extracellularly and thereby subjected to controls by powerful homeostatic machinery in the host, such as degradation by DNase I. Released NETs may be exposed to enzymatic digestion or enter general circulation where they can be detected. To determine if the levels of cell-free DNA in blood were associated with OO infection, which could be related to entry of OO-induced NETs into circulation, we evaluated the cell-free DNA concentration in the sera of uninfected controls and cattle infected with *O*. *ostertagi* for 15 or 29 days. Day 0 samples were collected immediately prior to infection. Serum DNA concentration did not seem to correlate to infection status and did not significantly differ between control and infected animals at days 15 and 29 post-parasite challenge (Fig. 4A). In addition, neutrophils from OO-infected cattle were able to form NET in response to stimuli (data not shown). Furthermore, the cell-free DNA concentration in sera was lower by approximately 2 log than those detected in the supernatant from stimulated neutrophils (Fig. 2). Indeed, the serum DNA was extracellular and sensitive to DNase I treatment, similar to those present in supernatants (Fig. 2B). Therefore, cell-free DNA levels in serum do not appear to be affected by *O*. *ostertagi* infection, suggesting that soluble DNA of NETs may not exit the infection sites, or be released but rapidly diluted or cleared^48^.

### Bovine neutrophils do not produce detectable ROS following OO extract exposure

**Figure 5:**
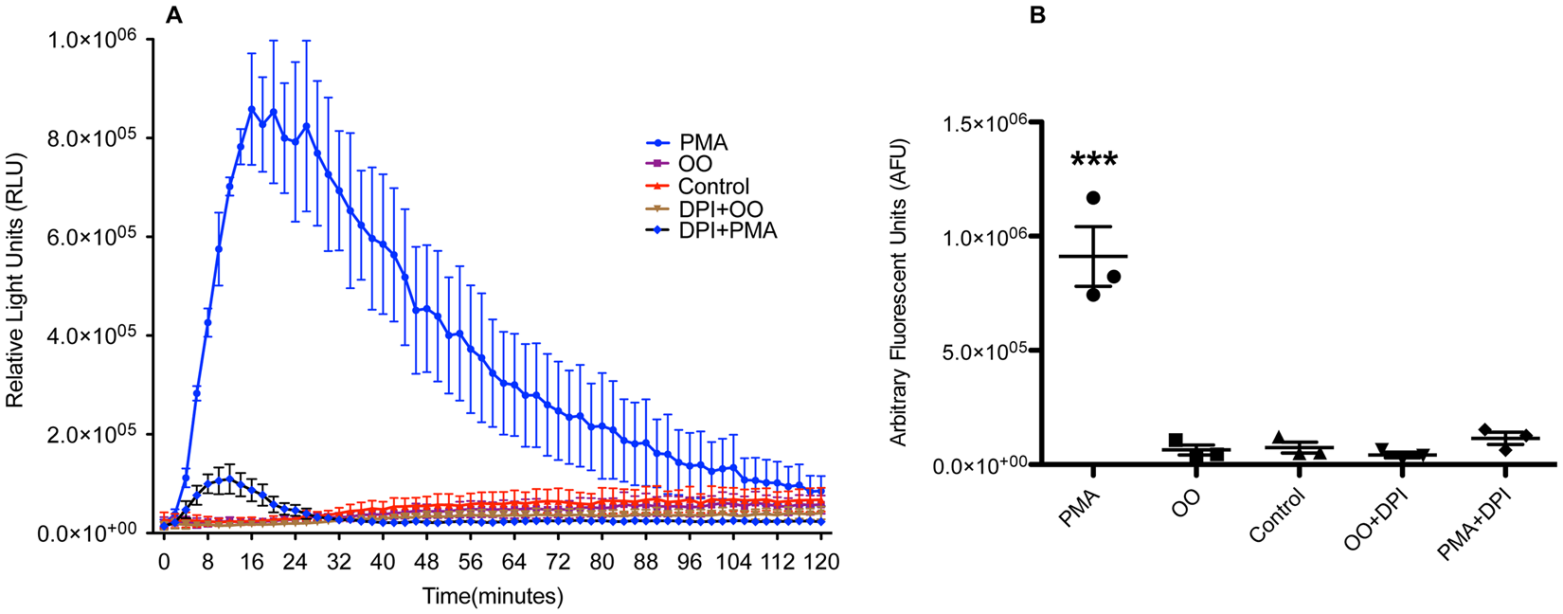
Reactive oxygen species (ROS) production by neutrophils measured by luminol-enhanced chemiluminescence assay. (**A**) Kinetics of ROS release by neutrophils under treatments over a period of 2 hours. (**B**) Total ROS production measured by area under each curve for the whole period of 2 hours. Experiments were performed in triplicate using cells from three cattle. Data are expressed as mean +/− SEM of triplicates. Data were analyzed by One-way ANOVA with Newman-Keuls Multiple Comparison Test. Data were representative of two experiments with similar results.

The NADPH oxidase complex mediates neutrophil production of ROS. Inhibition of NET release following incubation with the NADPH oxidase inhibitor DPI confirmed the importance of this enzyme in OO extract-induced NET formation. We speculated that ROS would be easily detected in the supernatant of neutrophils following stimulation with PMA, LPS and OO extract, based on Figs 2, 3. Supporting the dependence of ROS on NADPH oxidase complex, neutrophils treated with the inhibitor DPI showed significant reductions in ROS production following PMA stimulation (*p* ≤ 0.01, Fig. 5A,B). Surprisingly, OO extract did not induce significant production of ROS by neutrophils at any point over the 2 h stimulation period, compared to the strong ROS response seen in PMA-stimulated neutrophils (Fig. 5A). Similarly, when measured as total ROS released over time (the area under each curve), ROS was undetectable in the supernatant of OO extract-treated neutrophils (*p* ≥ 0.05, Fig. 5B); additionally, the ROS scavenger Vitamin C^49^ did not affect OO-induced NET release (Supplementary Fig. 1), suggesting that ROS may not be involved in the OO-induced NET formation. Taken together, these results indicate that ROS may be dispensable in OO extract-induced NET formation by neutrophils, and suggest that the lack of ROS production may partially contribute to the failure of parasite control, which warrants further investigation.

**Figure 6:**
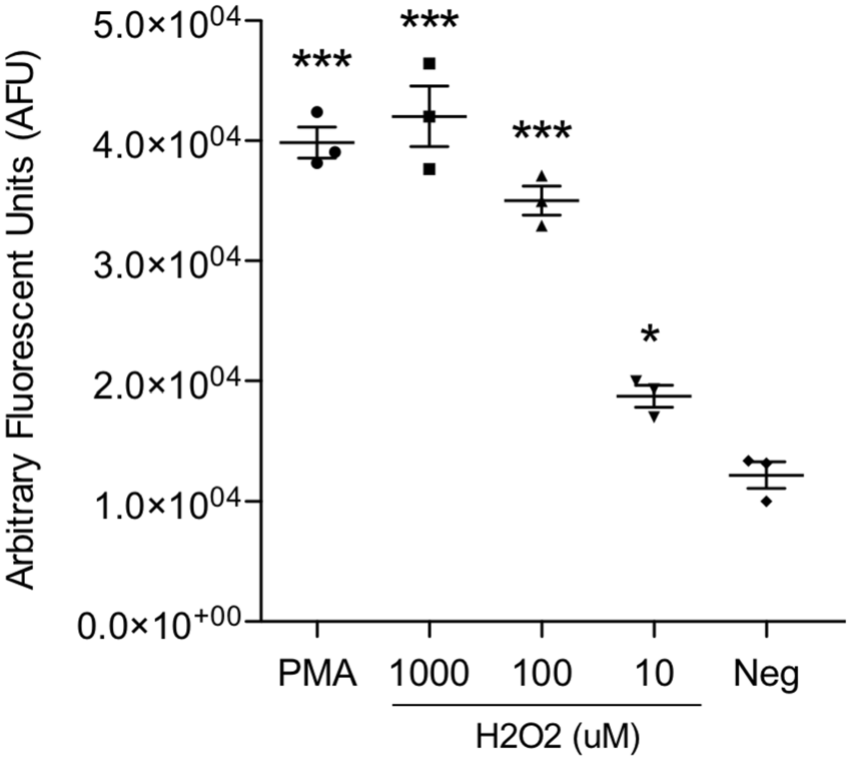
Hydrogen peroxide (H2O2) induces DNA release by bovine neutrophils dose-dependently. Experiments were performed in triplicate using cells from three cattle. Data were expressed as mean +/− SEM. Data were analyzed by one-way ANOVA with Newman-Keuls Multiple Comparison Test. Asterisks represented significance compared with negative control. Data were representative of two experiments with similar results.

Given the lack of ROS during OO extract-induced NET formation, it is important to know if bovine neutrophils were able to release NETs in response to ROS products. To test this possibility, hydrogen peroxide (H_2_O_2_) was used as an exogenous form of ROS in a range of concentration used in previous reports. Neutrophils exposed to varying concentrations of H_2_O_2_ displayed a significant, dose-dependent NET release (*p* ≤ 0.01, Fig. 6), equivalent to that of PMA-treated neutrophils, which was not affected by addition of DPI or PMA (data not shown). Therefore, despite the lack of detectable ROS production by neutrophils stimulated by OO extract and LPS, ROS products (H_2_O_2_) were able to directly stimulate NET formation by neutrophils, suggesting high responsiveness of bovine neutrophils to inflammatory mediators, such as ROS, that could lead to NET formation.

### OO extract-induced NET formation is not bovine-specific

**Figure 7:**
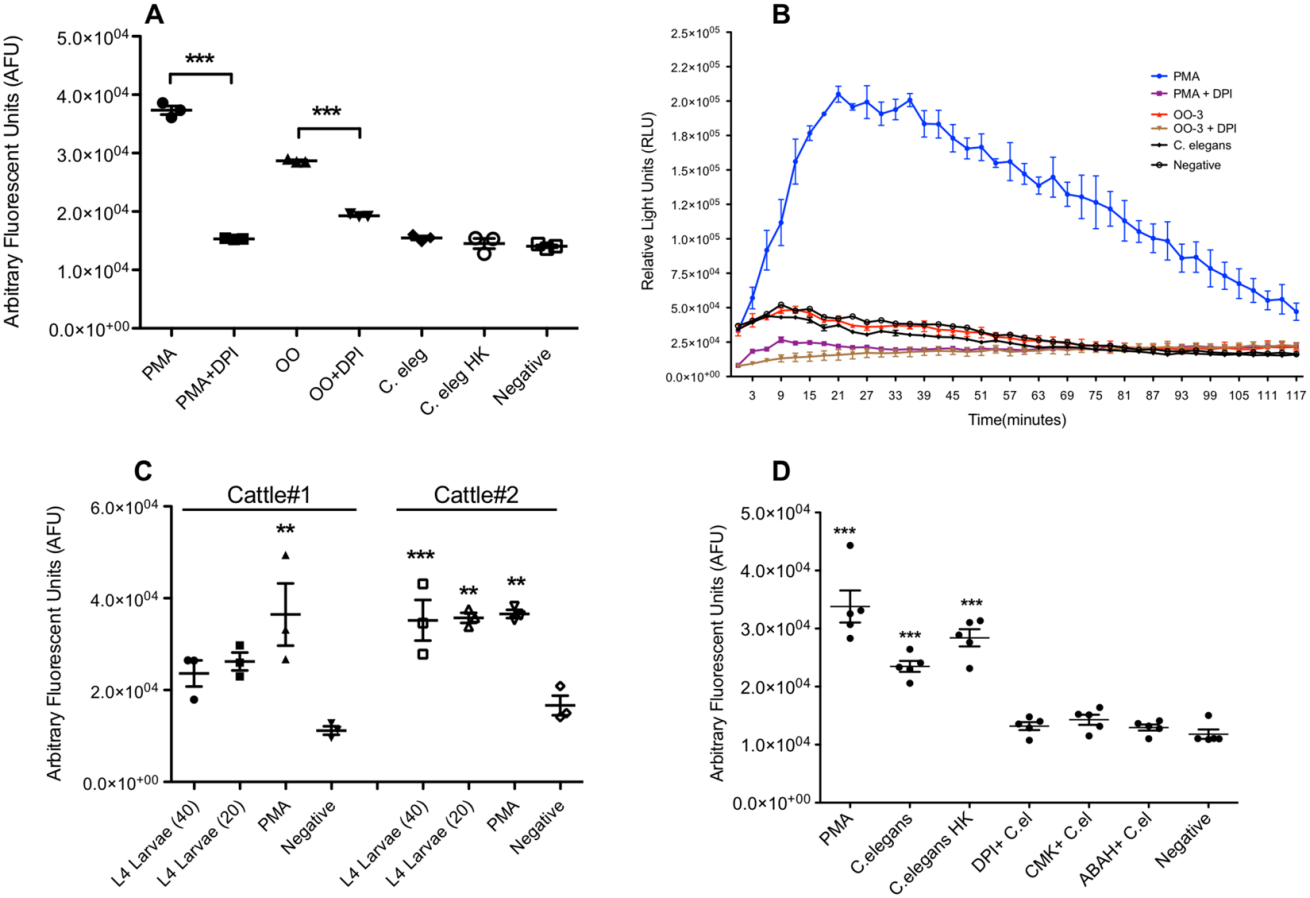
The induction of DNA release by neutrophils is a conserved function of the nematodes. Bone marrow-derived mouse neutrophils stimulated with *O. ostertagi* extract (OO) were assayed for DNA release 3 hours post-treatment (**A**) or ROS production over a period of 2 hours using luminol-enhanced chemiluminescence assay. (**C**,**D**) DNA release by bovine neutrophils from two cattle (**C**) or five cattle (**D**) following stimulation with live L4 Ostertagia larvae (**C**), or live *C. elegans* (**D**). Data were expressed as mean +/− SEM. The data in (**A**,**B**,**D**) represent two independent experiments in triplicates. C.el: live *C. elegans*. HK: heat-killed. Data were analyzed by One-way ANOVA with Newman-Keuls Multiple Comparison Test.

*O*. *ostertagi* is a nematode parasite that completes the parasitic developmental stages most commonly in cattle, and to a less extent in smaller ruminants such as sheep and goats, and does not establish infection or cause disease in rodents. However, OO extract was reported to influence mouse immune cell activity. To assess if *O*. *ostertagi* elicited NET formation in neutrophils from mice, bone marrow neutrophils from C57BL/6 mice were stimulated with OO extract and their ability to release NETs was examined. Interestingly, there was a significant increase in NET release by OO extract-treated mouse neutrophils (*p* ≤ 0.001, Fig. 7A), which was inhibited by the NADPH oxidase inhibitor DPI (*p* ≤ 0.001). However, this inhibition was not complete, unlike in bovine neutrophils (Fig. 3). In addition, *C*. *elegans*, a lab adaptive strain of a soil nematode, was not able to induce murine neutrophils to form NETs (Fig. 7A). Consistent with the bovine neutrophil results, neutrophils from mice also did not produce ROS following OO extract stimulation (Fig. 7B), further suggesting that *O*. *ostertagi* mediates NET release independent of ROS production in both bovine and mouse neutrophils. Therefore, these data indicate that NET release by neutrophils is a conserved defense response across mammalian species, possibly mediated by broad host-pathogen pattern recognition mechanisms^50^, or other relatively conserved components such as lectin.

### Bovine neutrophils release NETs in response to live free-living and parasitic nematodes

To reconcile whether whole parasites could induce NET formation, and a possible conserved mechanism across nematodes, live L4 stage *O*. *ostertagi* and *C*. *elegans* of mixed stages were tested for NETs induction in bovine neutrophils *in vitro*. Despite some variation among individuals, neutrophils co-cultured with either 20 or 40 per well live L4 *Ostertagia* larvae released significantly higher levels of NETs compared to unstimulated controls in both cattle (p < 0.05, Fig. 7C). In addition, the level of NETs induced by live L4 *Ostertagia* larvae was similar to the those of PMA stimulation in Cattle#2 but not Cattle#1 (Fig. 7C). *C*. *elegans*, a free-living nematode distantly related to most livestock GI nematodes, was chosen to compare against the parasite *O*. *ostertagi*. Unexpectedly, bovine neutrophils exposed to live worms of *C*. *elegans* showed significant induction of NET release (*p* ≤ 0.01, Fig. 7D), albeit not as large as those in PMA*-*stimulated neutrophils. *C*. *elegans-*induced NET release was independent of viability of the worms, as heat-killed worms (60 °C, 60 min) induced similar NET release (Fig. 7D). Inhibition experiments confirmed that these NETs by bovine neutrophils in response to *C*. *elegans* were also dependent upon NE, MPO, and NADPH oxidase (*p* ≤ 0.01, Fig. 7D). As such, bovine neutrophils appear to form NETs in response to different nematodes, and *O*. *ostertagi* may induce NETs in multiple host species, suggesting NET formation is a conserved defensive mechanism against a broad spectrum of pathogens including nematodes.

## Discussion

Parasite-induced NET formation has been extensively studied in humans and mice. However, few of the studies have focused on the effects of nematodes^51,52^. Bovine neutrophils can release NETs, upon stimulation from nematode or non-nematode parasites^18,22–25,27,32^. In this report, we provide the first evidence of a NET response elicited by *O*. *ostertagi*, one of the most detrimental GI nematode parasites to the cattle industry^53,54^. OO extract can induce bovine neutrophils to form NETs, which are dependent on MPO, NE, and NADPH oxidase, proteins previously reported to be involved in parasite-dependent NET formation in cattle^19,22,23,34^. In addition, live OO L4 larvae induced significant NET release. Unexpectedly, *C*. *elegans*, a lab adaptive strain of soil nematode, was also able to cause NET formation in bovine neutrophils. Moreover, neutrophils from cattle and mice form NETs in response to OO extract, suggesting possible common mechanisms in NET formation shared by mammalian species in responding to nematodes.

In infected abomasal mucosa, the parasite OO concentration should be relatively low, and there is a possibility of direct interaction of parasites with immune cells, especially at the start of acute OO infection. To simulate some of these *in vivo* situations, we first examined whether bovine neutrophils would respond to low concentrations of adult stage OO extract. The capacity of bovine neutrophils to release significant levels of NETs in response to low concentrations of OO extract suggests that this response may be viable *in vivo*, where the actual abundance of parasite antigen can be quite low. To assay whether the intact parasite could induce NETs, live L4 stage *O*. *ostertagi* were co-cultured with bovine neutrophils, resulting in a NET response similar to OO extract alone. This suggests that *O*. *ostertagi*, in either soluble extract or live intact organism, can induce NETs. Despite the fact that the functional role of neutrophils in nematode infection has just begun to be unfolded^55^, there are several recent reports on rapid recruitment of neutrophils to the site of infection by *Strongyloides stercoralis*^56^ or *Heligmosomoides polygyrus*^57^. Such a recruitment may be resulted from tissue injury caused by migrating larvae and parasite-derived chemotactic factors^55,58^. The *in vivo* effect of this parasite on NET release should be confirmed in future studies.

To explore the possibility that the bovine NET response might be deployed against other nematodes and not specific to *O*. *osteragi*, the non-parasitic nematode *C*. *elegans* was added to our experiments. Interestingly, and despite its lack of pathogenicity, *C*. *elegans* also induces bovine neutrophils to form NETs following stimulation. The increasing reports of NETs from various species in response to different microbes reinforces the idea of NETs being an ancient and conserved aspect of the innate immune system shared among vertebrates and plants^59,60^. Still, innate immunity differs among species. In addition, OO soluble extract contains specific immunoregulators, such as lectin^61^. Interestingly, some members of the lectin family, such as C-type lectin receptor, Mincle, do in fact induce NETs in mouse neutrophils^62^. In addition, P-selectin, another member of the lectin family, is directly involved in NET formation in mice^63^. Therefore, NET release by murine neutrophils in response to OO soluble extract could be induced or enhanced by the presence of additional immune-triggering components, such as lectin, which is conserved across many species^64^.

Given the heterogeneity of the soluble extract, multiple mediators present in the extract could be independently or synergistically initiating the NET response utilizing different pathways or receptors, such the various pattern recognition receptors present on the surface or in the endosomes of neutrophils (e.g. TLRs)^65^. In the case of the L4 larvae and *C*. *elegans*, in addition to the possible response to size^66^, there may also be molecular structures present on the surfaces of these worms that neutrophils are recognizing, and the possibility of such surface molecules being present in the soluble extract cannot be discounted. For example, various *Candida albicans* cell surface components are capable of inducing NETs via various receptors including TLRs, members of C-lectin family (Dectin-1), and complement receptors (CD11b/CD18, Mac-1)^67^. It is possible that multiple signals are simultaneously mediating the *O*.*ostertagi* response, potentially indicating redundant mechanisms for NET formation, as redundancy is common in immunological functions^68,69^.

**Figure 8:**
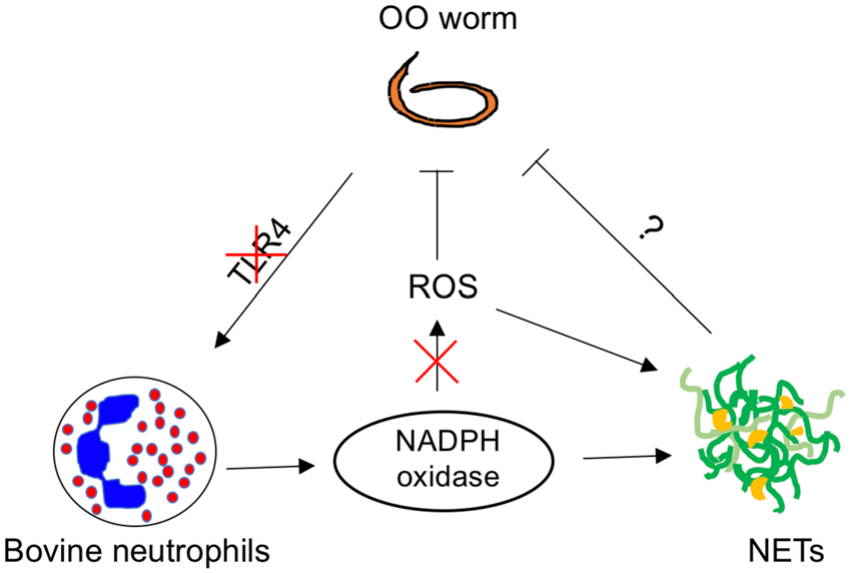
Bovine neutrophils form NETs in response to *O. Ostertagi*. This NETosis is dependent of NADPH, but may not involve stimulation through TLR4, or production of ROS.

Previous reports on parasite-mediated NET release have shown the importance of NE, MPO, and NADPH oxidase. Using chemical inhibitors, we have shown that these proteins are also crucial for the *O*. *ostertagi-*mediated NET response. Inhibition of NADPH oxidase with DPI completely abrogated the formation of NETs following *O*. *ostertagi* stimulation, confirming the importance of this pathway even though the main function of NADPH oxidase is the production of ROS^70^ (Fig. 8). However, there was no detectable release of ROS throughout the assay following *O*. *ostertagi* stimulation (Fig. 8). Reports of NADPH oxidase or ROS-independent NET pathways do exist^71,72^, with many demonstrations of mitochondrial-derived ROS triggering NETs^73–75^. While it is difficult to exclude the possibility of a NADPH oxidase-independent pathway to *O*. *ostertagi*-mediated NET formation, the complete lack of ROS detected during stimulation diminishes the likelihood of a mitochondrial-ROS pathway. Curiously, LPS is often considered an inducer of NETs^30^, yet it is widely known that LPS alone is a poor stimulator of ROS^76^, which we also observed.

We have shown that both bovine and murine neutrophils demonstrated no measurable ROS response to *O*. *ostertagi* stimulation. This suggests a potential difference between the non-physiological PMA-induced NET pathway and the *O*. *ostertagi* pathway, a concern that has been raised previously regarding the use of PMA^39^. In addition, while DPI is the most widely used inhibitor of NADPH oxidase, DPI can affect various other cellular processes as well, particularly in the mitochondria^77^. Data resulting from use of DPI may require more cautious interpretation than has been previously considered and the discovery of specific alternative inhibitors should be actively sought.

The ability of ROS itself, independent of NADPH oxidase, to induce NETs has been reported previously in humans^14^ and chickens^78^. In the current work, we therefore assayed NET release in cattle following addition of exogenous ROS in the form of H_2_O_2_, which induced NETs, even in the presence of NADPH oxidase inhibitor, confirming that bovine neutrophils are also capable of forming NETs in the presence of ROS alone. Interestingly, it has been shown that certain organisms such as *Candida albicans* possess the ability to detoxify or degrade ROS utilizing superoxide dismutases (SOD) and catalases^79,80^ as an effective strategy against neutrophils. It is plausible that similar enzymes may be present within nematode worms such as *O*. *ostertagi*. Paradoxically, it has also been reported that *C*. *albicans* is able to produce its own ROS^47^, enough to rescue NET formation in CGD-isolated neutrophils. However, these explanations do not align with the demonstrated lack of discernable ROS production. In addition, the presence of the ROS scavenger Vitamin C did not affect OO-induced NET formation, further diminishing the role of ROS in OO-induced NET formation. Overall, our results suggest that the formation of NETs may be dependent upon multiple factors, of which ROS production is but one and may not be necessary (Fig. 8).

Our data clearly demonstrate the ability of *O*. *ostertagi* to induce NET formation, suggesting a potential role for NET formation in the innate immune response of cattle to the parasite *O*. *ostertagi*. Further, the response of NET formation is not specific to live, fully intact parasite but can also be induced with parasite antigen alone. Surprisingly, the *O*. *ostertagi-*mediated NET response appears to be independent of ROS, but requires NADPH oxidase activity (Fig. 8). While this result is difficult to interpret, a plausible hypothesis is that multiple pathways are involved in the NET response to *O*. *ostertagi*, which is clearly distinct from the PMA-induced NETs previously studied. In addition, it is certain that the *in vivo* context presents additional complexity compared to the simplified, single cell type models. The contribution of cytokines, chemokines, and interactions with other cells in the local milieu can alter the response as well as outcome, which warrants further investigations. While the actual ability of NETs to capture or even kill *O*. *ostertagi* and its relevance *in vivo* remain unclear, we nevertheless conclude that *O*. *ostertagi* is able to induce NETs *in vitro* (Fig. 8), and suggest that NETs can be a potential immune defense mechanism by against infection.

## Methods

### Cattle

The Wye Angus herd is a closed herd maintained by the Wye Research and Education Center, University of Maryland Experimental Station (Queenstown, MD)^81^. The steers The steers were maintained on the pasture of orchard grass, alfalfa, or clover, and fed with alfalfa, and bailage in winter^82^. Helminth-free Holstein steers were raised and maintained indoors on concrete slab since birth on the campus of Beltsville Agricultural Research Center (BARC), Beltsville, MD. Jugular venous blood was obtained for neutrophil and serum isolation. Animal Care and Use Protocols were approved by both the BARC (#16–019) and UMD (R-FEB-18-06) Institutional Animal Care and Use Committees. All methods were performed in accordance with the relevant guidelines and regulation.

### Parasite propagation and parasitic antigen preparation

*O*. *ostertagi* adult worms and stage four larvae (L4) were propagated in helminth-free calves as described previously^83^. Briefly, 4–6 months old, helminth-free Holstein steers were inoculated with a bolus dose of *O*. *ostertagi* L3 on Day 0 and euthanized on Day 9 for L4 larvae or Day 21 for adult worm, and the abomasum and abomasal content were collected for parasite isolation. Parasites were collected from the abomasal tissue using the Baermann technique (L4) or from the abomasal content using the gel migration method (adult). Following collection by Baermanization, live L4 larvae were washed 3 times in cell culture medium containing penicillin, streptomycin, and fungizone in 5-time strength. To remove antibiotics, the larvae were washed 5 times with antibiotic-free cell culture medium. Adult parasites were washed 2 times with cold PBS and homogenized in cold PBS on ice at maximum speed for five 15-sec pulses using a Polytron homogenizer (Brinkmann Instrument, Westbury, NY). The homogenate was centrifuged at 20,000 *g* for 30 min at 4 °C and soluble extract (hereafter referred to as “OO extract”) was stored at −20 °C prior to experimental use.

For the *Caenorhabditis elegans* (*C*. *elegans)* experiments, a mixed population (early stage larvae to adult) of soil nematode *Caenorhabditis elegans* was grown at 20 °C in axenic liquid mCeHR-2 medium supplemented with 20 μM hemin^84^. Both live and heat-killed (60 °C, 60 min) worms were centrifuged at 800 *g* and washed twice with PBS before being resuspended in RPMI media^85^.

### Bovine neutrophil isolation

Jugular vein blood was collected from cattle using vacutainers containing EDTA or no additive (Becton Dickinson Vacutainer Systems, Franklin Lakes, NJ). Neutrophils were isolated as previously described^84^ with minor modifications; briefly, blood was transferred to 15 mL conical tubes (Fisher Scientific, Pittsburgh, PA, USA) and centrifuged for 20 min at 1,000 *g* at 4 °C. Following centrifugation, the plasma, buffy coat, and one-third of the red blood cell pellet were discarded. The remaining cells were resuspended in 5 mL ammonium-chloride-potassium (ACK) lysis buffer to remove red blood cells. The cell suspension was gently mixed and incubated for 5 min at room temperature (RT). The solution was then centrifuged for 10 min (200 *g* at 4 °C) and the supernatant was decanted. The pellet was washed with 15 mL of calcium- and magnesium-free PBS (CMF-PBS) and centrifuged for 5 min (850 *g* at 4 °C). For complete red blood cell lysis, ACK treatment was repeated. Cells were then washed twice with 15 mL of CMF-PBS and centrifuged for 5 min (850 *g* at 4 °C). After the final wash, the pellet was resuspended in 1 mL of RPMI 1640 lacking phenol red (Gibco, Fisher Scientific), and neutrophil concentrations were measured using the trypan blue exclusion method on a hemocytometer.

### Mouse neutrophil isolation

Neutrophils were isolated from bone marrow of tibias and femurs of adult mice by density gradient centrifugation as described previously^86–88^ and resuspended in phenol red-free RPMI 1640 medium containing 2% FBS.

### NET Quantification

Neutrophils were re-suspended in RPMI 1640 medium containing 2% FBS and lacking phenol red. Cells were deposited in triplicate into 96-well flat-bottom plates (Nunc, Fisher Scientific) and incubated for 30 min (preincubation) at 37 °C and 5% CO_2_ prior to stimulation. Cells were then stimulated for up to 3 hours with OO extract (3 µg/mL) or Toll-like receptor 4 (TLR4) ligand LPS (100 ng/mL, InvivoGen, San Diego, CA) in a final volume of 200 µL per well_._ Dose dependency of OO extract was evaluated using further dilutions (1:10 and 1:100) in RPMI medium. For live worm experiments, neutrophils were cultured with either *C*. *elegans* (20 or 40 worms/well) or *O*. *ostertagi* L4 larvae (40 worms/well) in triplicate.

Following stimulation, micrococcal nuclease was added (5 U/well, New England Biolabs, Ipswich, MA, USA) and incubated for 15 min. Samples were centrifuged (800 *g*, 5 min) and the supernatants (100 μL/well) were transferred to a black 96-well flat-bottom plate (Nunc). The samples were stained with the fluorescent DNA dye Sytox Green (5 µM final concentration, Invitrogen, Carlsbad CA, USA) and incubated at RT in the dark for 10 min^89^. NET formation was quantified in arbitrary fluorescent units (AFU) by spectrofluorometric analysis with an excitation wavelength of 485 nm and an emission wavelength of 525 nm using an automated plate reader (Biotek, Winooski, VT, USA). For negative controls, unstimulated neutrophils in regular RPMI medium lacking phenol red were used. Neutrophil stimulation with phorbol 12-myristate 13-acetate (PMA; Sigma, St. Louis, MO, USA 100 nM final concentration) served as a positive control^19^.

#### Inhibition Assays

Specific inhibitors described previously were used for blockage of NET formation and have been described previously^23,34,40,46,90–94^. The following inhibitors were used: the NE inhibitor Suc-Ala-Ala-Pro-Val chloromethyl ketone (CMK; 1 mM final concentration, Sigma), NADPH oxidase (NOX) inhibitor diphenylene iodonium (DPI; 10 μM final concentration, Sigma), the MPO inhibitor 4-Aminobenzoic acid hydrazide (ABAH; 100 μM final concentration, Sigma), TLR4 signaling inhibitor CLI-095 (1 μg/mL final concentration; InvivoGen), the TLR4 signaling inhibitor polymixin B (100 μg/mL final concentration; InvivoGen, San Diego, CA), the PKC inhibitor Bisindolylmaleimide I (310 nM final concentration, Cayman Chemical, Ann Arbor, MI), and the ROS scavenger inhibitor ascorbic acid (Vitamin C) (200 μM final concentration, Cayman Chemical, Ann Arbor, MI). Cells were pre-incubated with inhibitors for 30 min at 37 °C prior to stimulation as described above^23,34^.

### Visualization of NETs and Detection of NET-associated proteins

Isolated neutrophils (3 × 10^5^) were seeded on 13 mm round glass coverslips pre-treated with poly-L-lysine (Sigma) in 24-well plates and were allowed to adhere for 30 min at 37 °C^95^. Cells were then stimulated as described previously for up to 3 hours. Following treatment, coverslips were washed with PBS and fixed in 2% paraformaldehyde for 15 min at RT. Coverslips were then washed 3 times with PBS and blocked with 2% BSA (Sigma) for 30 min to prevent non-specific binding. To detect histone or NE, coverslips were incubated with anti-histone (H3) antibody (Fisher Scientific) at 1:1000 or anti-NE antibody (Abcam, Cambridge, MA, USA) at 1:200 for 1 h at room temperature (RT). Following first antibody incubation, coverslips were washed twice with PBS and incubated 30 min at RT with anti-mouse IgG-PE (Biolegend, San Diego, CA, USA) diluted at 1:500 in blocking buffer. Coverslips were subsequently stained with Sytox Green (1:1000, 15 min), washed twice with PBS, and mounted on glass slides using anti-fade mounting buffer (Fisher Scientific). Images were taken using a laser scanning confocal microscope (Zeiss LSM 510 system, Thornwood, NY, USA).

### Measurement of DNA in sera from OO-challenges and uninfected control cattle

Cattle were infected with OO L3 and venous blood samples were collected at days 0, 15 and 29 post infection. Serum was carefully harvested, without pigment contamination^96^. Serum sample (10 µL) was added to 90 µL of PBS, followed by addition of 100 uL of Sytox Green (1:200) per well in a black 96-well flat bottom plate (Nunc) and incubated in the dark for 15 minutes at RT. Fluorescence was quantified as described in “NET Quantification” using a spectrofluorometer.

### Chemiluminescent measurement of ROS production

ROS production was measured by chemiluminescence as described previously^14,97^. Neutrophils (1 × 10^5^) were resuspended in RPMI 1640 containing 11 mM HEPES, 55 mM Luminol (Fisher), and 1.2 U/mL horse radish peroxidase (Sigma). Ninety µL of the cells was then plated in a white 96-well flat bottom microplate (Nunc) and subsequently stimulated with 10 µL of stimuli as described in “NET quantification”. Chemiluminescence was recorded for 1 s per well every 2 min for 30 min prior to addition of stimuli, and for an additional 2 h using an automated plate reader set to 37 °C.

### Statistical Analysis

Statistical analysis was performed with Prism 5 (GraphPad Software, Inc., La Jolla, CA), with specific details described in figure legends. Overall, all data have passed the Kolmogorov–Smirnov normality test. All data were analyzed by one-way ANOVA with Newman-Keuls Multiple Comparison Test. Asterisks indicate statistical significance. *P < 0.05; **P < 0.01; ***P < 0.001.

## Electronic supplementary material

Supplementary Figure 1
