# Supplementary material for: Bovine neutrophils form extracellular traps in response to the gastrointestinal parasite Ostertagia ostertagi
Source: Sci Rep. 2018 Dec 4;8:17598. doi: 10.1038/s41598-018-36070-3 (PMC6279769; doi:10.1038/s41598-018-36070-3)
Supplement: Supplementary file 1 — Supplementary Figure 1 [file 41598_2018_36070_MOESM1_ESM.pdf]

Bovine neutrophils form extracellular traps in response to the gastrointestinal parasite *Ostertagia ostertagi*

Jonatan Mendez[1], Donglei Sun[2], Wenbin Tuo[3] and Zhengguo Xiao[1]

[1]Department of Avian and Animal Sciences, University of Maryland, College Park, MD 20742;

[2]Department of Veterinary Medicine, University of Maryland, College Park, MD 20742;

[3]Animal Parasitic Diseases Laboratory, USDA/ARS, Beltsville, MD 20705;

*Corresponding authors

Wenbin Tuo, wenbin.tuo@ars.usda.gov; Zhengguo Xiao, xiao0028@umd.edu

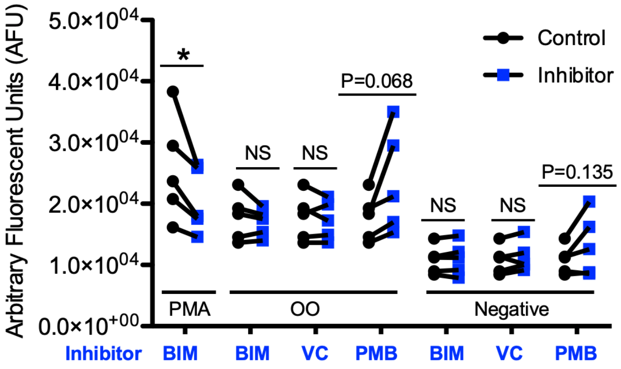

Supplementary Figure. 1. Involvement of PKC, ROS and LPS in OO induced NETs. A. Inhibition of PKC with BIM, ROS with scavenger VC, and LPS with PMB. Data were expressed as paired comparisons of NETosis between control (black) and inhibitors (blue) of 5 cattle samples. Data were analyzed by paired Student's *t* test. BIM: Bisindolylmaleimide I. PMB: Polymyxin B. VC: vitamin C.
